# Supplementary material for: Spliceosome-Mediated Pre-mRNA trans-Splicing Can Repair CEP290 mRNA
Source: Mol Ther Nucleic Acids. 2018 Jun 27;12:294–308. doi: 10.1016/j.omtn.2018.05.014 (PMC6023944; doi:10.1016/j.omtn.2018.05.014)
Supplement: Document S1. Figure S1 [file mmc1.pdf]

OMTN, Volume *12*

Supplemental Information

Spliceosome-Mediated Pre-mRNA

trans-Splicing Can Repair *CEP290* mRNA

Scott J. Dooley, Devin S. McDougald, Krishna J. Fisher, Jeanette L. Bennicelli, Lloyd G. Mitchell, and Jean Bennett

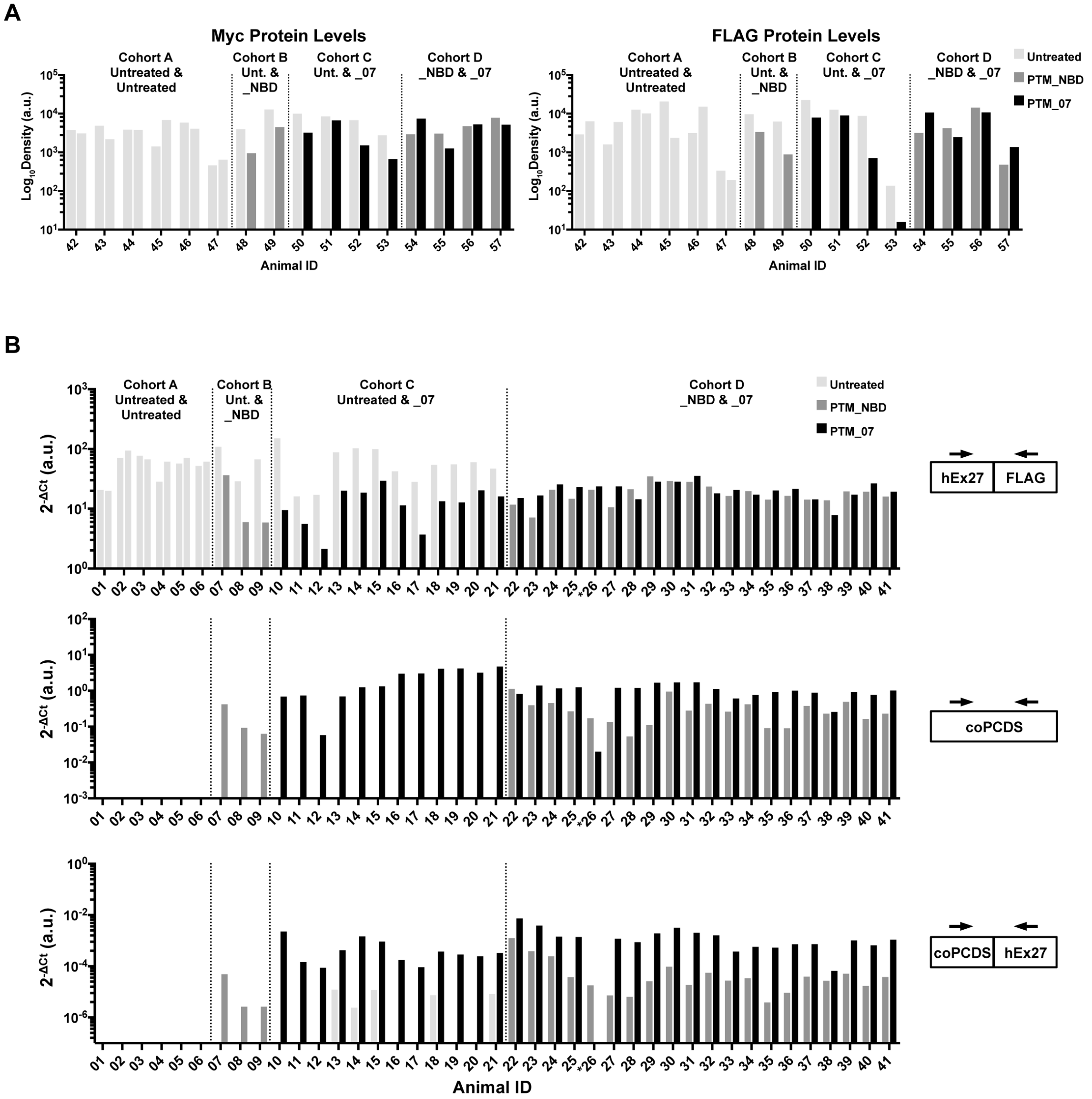

Figure S1. Individual treated murine samples by contralateral eye.

(A) Densitometry quantification of western blot images of Myc and FLAG at 24.3 kDa from protein extracts of whole eyes of mini-*CEP290* mice. Eyes were treated by sub-retinal injection with 5' PTMs encoded in AAV serotype 7m8 capsids. Samples are grouped by contralateral eye treatments per animal and treatment cohorts are separated by vertical dashed lines. Values were standardized to α-Tubulin and logarithm values were utilized to compare substantially different values between mice. A.U., arbitrary units.

(B) Quantitative PCR from cDNA generated by RNA extracts from whole eyes of mini-*CEP290* mice. TaqMan probes were designed to the junctions of *homo sapiens* exon 27 and FLAG to detect total expression of the mini-gene (*top*), to a region within the PCDS to detect total expression of the PTM (*center*), or to the novel junction of codon-optimized *CEP290* PCDS and *Homo sapiens CEP290* exon 27 (*bottom*). Injection of animal 26 with PTM_07, indicated by asterisk (*) did not yield a visible bleb and concurrently no *trans*-splicing was detected in this sample. Samples are grouped by contralateral eye treatments per animal and treatment cohorts are separated by vertical dashed lines. Ct values were standardized to the β-2-microglobin control gene to generate ΔCt values. A.U., arbitrary units.
