# Supplementary material for: Spliceosome-Mediated Pre-mRNA trans-Splicing Can Repair CEP290 mRNA
Source: Mol Ther Nucleic Acids. 2018 Jun 27;12:294–308. doi: 10.1016/j.omtn.2018.05.014 (PMC6023944; doi:10.1016/j.omtn.2018.05.014)
Supplement: Document S2. Article plus Supplemental Information [file mmc2.pdf]

Molecular Therapy
Nucleic Acids
Original Article

Spliceosome-Mediated Pre-mRNA *trans*-Splicing Can Repair *CEP290* mRNA

Scott J. Dooley,[1] Devin S. McDougald,[1] Krishna J. Fisher,[1] Jeanette L. Bennicelli,[1] Lloyd G. Mitchell,[2] and Jean Bennett[1]

[1]Center for Advanced Retinal and Ocular Therapeutics, Department of Ophthalmology, Perelman School of Medicine, University of Pennsylvania, Philadelphia, PA 19104, USA; [2]RetroTherapy, Bethesda, MD 20814, USA

Ocular gene therapy with recombinant adeno-associated virus (AAV) has shown vector-mediated gene augmentation to be safe and efficacious in the retina in one set of diseases (retinitis pigmentosa and Leber congenital amaurosis (LCA) caused by *RPE65* deficiency), with excellent safety profiles to date and potential for efficacy in several additional diseases. However, size constraints imposed by the packaging capacity of the AAV genome restrict application to diseases with coding sequence lengths that are less than 5,000 nt. The most prevalent retinal diseases with monogenic inheritance are caused by mutations in genes that exceed this capacity. Here, we designed a spliceosome mediated pre-mRNA *trans*-splicing strategy to rescue expression of *CEP290*, which is associated with Leber congenital amaurosis type 10 (LCA10) and several syndromic diseases including Joubert syndrome. We used this reagent to demonstrate editing of *CEP290* in cell lines *in vitro* and *in vivo* in a mini-gene mouse model. This study is the first to show broad editing of *CEP290* transcripts and *in vivo* proof of concept for editing of *CEP290* transcripts in photoreceptors and paves the way for future studies evaluating therapeutic effects.

INTRODUCTION

Leber congenital amaurosis (LCA) is the most severe subset of the set of diseases classified as retinitis pigmentosa, affecting 1 in 100,000 people with nearly 20 identified disease-associated genes. LCA is typically diagnosed in infants with severely abnormal vision through finding of non-recordable electroretinograms. Children with LCA have trouble seeing in dim light (nyctalopia), have reduced visual resolution (visual acuity) and peripheral vision (visual fields), and often have abnormal rotatory eye movements (nystagmus). Whatever poor vision they have early in life gradually disappears due to the degenerative component of the disease.[1] Amelioration of one form of early-onset retinal degeneration caused by the gene *RPE65* has been demonstrated following sub-retinal administration of recombinant adeno-associated virus (AAV) encoding the complete *RPE65* coding DNA sequence.[2–4] Other forms of inherited retinal degeneration may be amenable to rescue following this strategy; however, the genes associated with the most prevalent forms of retinal disease exceed the DNA coding capacity of AAV.[5] Mutations in *CEP290* are designated as LCA type 10 (LCA10), and the complete coding DNA sequence is 7,440 nt (GeneBank: NM_025114.3). Therefore, it is necessary to develop a gene therapy strategy that can correct a broad range of mutations in large genes such as *CEP290* with AAV.

Most LCA10 patients harbor two mutations in *CEP290*, which are typically compound heterozygous. The most common mutation in LCA10 is a single nucleotide polymorphism of A to G within the intron between exons 26 and 27. The mutation is annotated as IVS26 c.2991+1655 A > G, hereafter IVS26, and generates a cryptic splice site leading to inclusion of a cryptic exon, known as exon X, which encodes an immediate stop codon (Figure 1A). Though fully differentiated, light-responsive photoreceptors generated from induced pluripotent stem cells have yet to be generated, optic cups generated from IVS26 homozygous patients show reduced CEP290 protein, shorter cilia, and other deficits compared to wild-type cell lines.[6] Additionally, other cell types, including primary fibroblasts, show similar phenotypes.[7]

Based on the prevalence of IVS26 within LCA10 patients and likely disease pathogenesis through haploinsufficiency, several strategies have been developed to specifically prevent inclusion of cryptic exon X. Several groups have targeted the RNA-directed endonuclease *Cas9* to the cryptic splice site to either alter the sequence or specifically use homology-directed repair to correct the mutation.[8,9] Anti-sense oligonucleotides have also been evaluated in several settings with IVS26 homozygous patient-derived cells and shown to reduce inclusion of exon X and restore CEP290 protein levels.[10] One concern is that the utility of the *Cas9* or anti-sense oligonucleotide methods directed at exon X may not be as high in compound heterozygous patients or in patients lacking the IVS26 mutation since the second allele would not benefit from therapy. Lastly, efforts have been made to break the coding DNA sequence of a large gene into fragments across multiple viral genomes.[11,12] When applied to LCA10, two AAV genomes are designed with either the 5′ or 3′ half of *CEP290* along with elements required to reconstitute the open

Received 14 December 2017; accepted 16 May 2018;
https://doi.org/10.1016/j.omtn.2018.05.014.

Correspondence: Jean Bennett, Center for Advanced Retinal and Ocular Therapeutics, Department of Ophthalmology, Perelman School of Medicine, 310 Stellar-Chance Laboratories, University of Pennsylvania, 422 Curie Boulevard, Philadelphia, PA 19104, USA.
E-mail: jebennet@pennmedicine.upenn.edu

 © 2018 The Author(s).
This is an open access article under the CC BY license (http://creativecommons.org/licenses/by/4.0/).

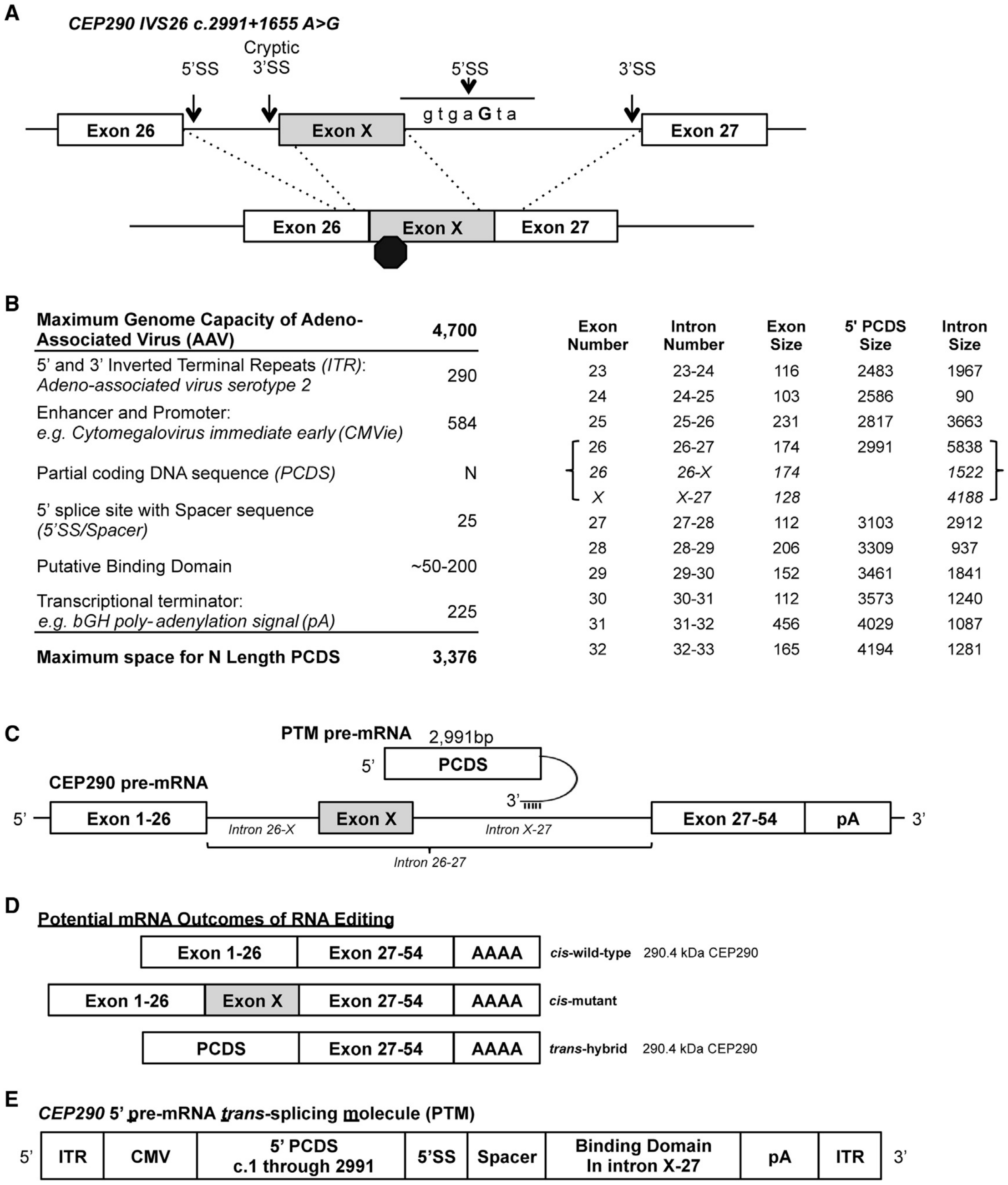

Figure 1. Strategy to Rescue *CEP290* through 5′ Pre-mRNA *trans*-Splicing

(A) Diagram of the most prevalent mutation in Leber congenital amaurosis type 10. This intron variant sequence (IVS) is an A-to-G transition that creates a canonical 5′ splice site (5′ SS). The novel splice site leads to inclusion of a cryptic exon, exon X. This cryptic exon encodes a premature stop codon leading to a truncated protein. (B) Left, computational analysis of required elements for a *trans*-splicing molecule and the size limitations of adeno-association virus. Right, analysis of *CEP290* exon and intron boundaries reveals the size of a 5′ PCDS when using a 5′ *trans*-splicing approach to target various introns. When exon X is included in the transcript, intron 26-27 is split into intron 26-X and intron X-27. A complete table for *CEP290* is included in Table 1. (C) Schematic of an approach to utilize a 5′ pre-mRNA *trans*-splicing molecule (PTM) to rescue mutations in *CEP290* that are located 5′ to intron X-27. The PTM transcript is bound via Watson-Crick base pairing to the pre-mRNA of a target via a sequence known as the binding domain, located at the 3′ end of the PTM, and that is reverse complementary to the target sequence. (D) Three potential splicing outcomes with *CEP290* IVS26 following introduction of a 5′ PTM: (1) joining of exon 26 to exon 27 from *cis*-splicing for the wild-type junction; (2) inclusion of exon X from *cis*-splicing (predominant mRNA

(legend continued on next page)

reading frame.[13] The limitations of this approach concern (1) efficiency—vectors of each genome must transduce the same cell and further, the genomes must recombine or undergo concatamerization in the proper orientation, and (2) potential immunogenicity—both fragments have the potential to produce partial gene products, which may trigger an immune response.

Delivery of AAV genomes encoding partial coding DNA sequences designed to *trans*-splice to endogenous pre-mRNA is also an attractive strategy. This strategy is akin to the multiple vectors strategy but differs in that a single viral genome is used that targets endogenous pre-mRNA transcripts to yield a hybrid exogenous-endogenous mRNA. Additionally, this strategy takes advantage of endogenous expression levels, which is a safety consideration since *CEP290* overexpression may cause toxicity.[7]

Trans-splicing was first shown to occur *in vitro*.[14,15] Since then, *trans*-splicing molecules have been demonstrated with 5′, 3′, or internal exon replacement.[16–18] Pre-mRNA *trans*-splicing molecules have been demonstrated to edit genes across many disease models including cystic fibrosis,[19–22] muscular dystrophy,[23–25] hypertrophic cardiomyopathy,[24,26] and retinitis pigmentosa 1.[17] Some efforts have also been made to apply *trans*-splicing to the treatment of cancer cells, including functional repair of p53 in colorectal cancer cells and hepatocellular carcinoma cells.[27,28] Here, we evaluated the potential of pre-mRNA *trans*-splicing to edit *CEP290* and demonstrated that this approach can be effective in yielding hybrid RNA both *in vitro* and *in vivo*.

RESULTS

Design of a *trans*-Splicing Strategy for CEP290

A strategy to rescue *CEP290* should cover as many disease-associated mutations as possible. Therefore, we wanted the maximum length of possible partial coding DNA sequence. We initially designed a hypothetical pre-mRNA *trans*-splicing molecule consisting of flanking inverted terminal repeats, the cytomegalovirus immediate-early enhancer and promoter, the partial coding DNA sequence whose maximum size we were to determine, a canonical 5′ splice site, 20 nt of intron spacer sequence, a putative binding domain, and a bovine growth hormone poly-adenylation signal sequence. After subtracting these elements from the maximum 4,700 nt of AAV, the maximum partial coding DNA sequence (PCDS) size for a *trans*-splicing AAV genome can be estimated to be between 3,000 and 3,500 nt (Figure 1B, left).

For a 5′ pre-mRNA *trans*-splicing molecule (PTM), the PCDS must end at the 3′ end of an exon to allow for a 5′ splice site. Therefore, PCDS size increases by the size of the exon proximal to a target intron. When plotted as a table, the PCDS size is the sum of all coding nucleotides upstream to the target intron. The first methionine of the open reading frame (ORF) for *CEP290* is encoded in the second exon. Therefore, the PCDS begins within exon 2 and continues to the stop codon in exon 54. We generated a table consisting of *CEP290* exon numbers, nucleotide length of exons and introns, and a tally of the coding nucleotides at each boundary (Table 1).

To rescue the most common mutations that cause LCA10, at minimum, a 5′ PTM must be targeted to a region downstream of exon X. By definition, exon X breaks intron 26-27 into the novel introns 26-X and X-27. We determined that intron X-27 would be 4,188 nt and would require a 5′ PCDS consisting of 2,991 nt (Figure 1B, right). We selected intron X-27 as the region to target and screen for putative binding domains (Figure 1C). Treatment of cells containing the IVS26 mutation with a *trans*-splicing AAV genome targeting intron X-27 would result in three predicted outcomes: (1) a fraction of transcripts splicing normally to generate a wild-type allele, (2) inclusion of exon X via *cis*-splicing leading to expected nonsense mediated decay, and (3) the *trans*-spliced hybrid of 5′ PCDS and endogenous exons 27–54 with the potential to generate wild-type protein (Figure 1D).

We then designed an AAV genome encoding the cytomegalovirus (CMV) promoter driving expression of a 5′ PTM containing coding nucleotides 1 through 2,991, which encodes Met1 through Glu997 p.(M1-E997) and added a cloning site for addition of a binding domain (Figure 1E).

Screening of Binding Domains in CEP290 Intron 26

The specificity of a PTM is conferred by the binding domain annealing to a target pre-mRNA through Watson-Crick base pairing. To identify a functional binding domain in intron X-27, we adapted a previously described fluorescence reconstitution assay.[29,30] In brief, the complete coding DNA sequence of the *Aequorea coerulescens*-derived *GFP* mammalian enhanced (codon-optimized) sequence was split between Glu112 and Val113 (GAG|GTG).[31] To screen binding domains for a 5′ *trans*-splicing strategy, the 5′ GFP PCDS (Met1 through Glu112) was cloned into a 5′ PTM reporter, while the 3′ GFP PCDS (Val113 through Stop240) was cloned downstream of the complete *CEP290* target intron 26-27 in a novel mini-gene plasmid. A *trans*-splicing event between the pre-mRNAs of the two plasmids would thus reconstitute the *GFP* ORF and result in GFP-positive cells (Figure 2A).

We evaluated the sequence of intron X-27 for frequently cutting blunt restriction enzymes with consensus sites in the target region.[32] This analysis revealed that a combination of RsaI and DraI was predicted to yield fragments between 50 and 300 nt, which has been described as the optimal size for binding domains[33] (Figure 2B). We then amplified intron X-27 from genomic DNA of a healthy donor and

species with IVS26 present); (3) joining of the 5′ PCDS to exon 27 from *trans*-splicing. Both outcomes 1 and 3 would result in full-length CEP290 peptide. (E) Illustration of an adeno-associated virus genome encompassing the therapeutically relevant *CEP290* 5′ *trans*-splicing molecule. Inverted terminal repeats (ITR) flank an expression cassette consisting of the cytomegalovirus promoter (CMV), a PCDS encoding the first 2,991 nt of the coding DNA sequence, a 5′ splice site (5′ SS), a spacer region, a binding domain that is reverse complementary to a region of intron X-27, and a polyadenylation signal sequence (pA).

Table 1. *CEP290* Exon and Intron Boundaries with Corresponding Partial Coding DNA Sequence

Exon No.	Exon Size	5′ PCDS	3′ PCDS	Intron Size	Intron Name
1	317	–	7,440	565	1-2
2	129	102	7,338	172	2-3
3	78	180	7,260	1,391	3-4
4	70	250	7,190	303	4-5
5	47	297	7,143	2,358	5-6
6	144	441	6,999	5,424	6-7
7	54	495	6,945	599	7-8
8	21	516	6,924	124	8-9
9	153	669	6,771	391	9-10
10	183	852	6,588	658	10-11
11	90	942	6,498	2,507	11-12
12	123	1,065	6,375	946	12-13
13	124	1,189	6,251	4,079	13-14
14	170	1,359	6,081	720	14-15
15	163	1,522	5,918	1,370	15-16
16	101	1,623	5,817	72	16-17
17	88	1,711	5,729	1,337	17-18
18	113	1,824	5,616	1,850	18-19
19	85	1,909	5,531	535	19-20
20	143	2,052	5,388	2,561	20-21
21	165	2,217	5,223	342	21-22
22	150	2,367	5,073	2,020	22-23
23	116	2,483	4,957	1,967	23-24
24	103	2,586	4,854	90	24-25
25	231	2,817	4,623	3,663	25-26
26	174	2,991	4,449	5,838	26-27
27	112	3,103	4,337	2,912	27-28
28	206	3,309	4,131	937	28-29
29	152	3,461	3,979	1,841	29-30
30	112	3,573	3,867	1,240	30-31
31	456	4,029	3,411	1,087	31-32
32	165	4,194	3,246	1,281	32-33
33	108	4,302	3,138	217	33-34
34	135	4,437	3,003	1,186	34-35
35	267	4,704	2,736	631	35-36
36	108	4,812	2,628	616	36-37
37	200	5,012	2,428	2,635	37-38
38	214	5,226	2,214	952	38-39
39	138	5,364	2,076	1,173	39-40
40	222	5,586	1,854	352	40-41
41	123	5,709	1,731	5,295	41-42
42	146	5,855	1,585	331	42-43
43	156	6,011	1,429	2,648	43-44
44	124	6,135	1,305	4,406	44-45
45	135	6,270	1,170	1,202	45-46
46	87	6,357	1,083	1,697	46-47
47	165	6,522	918	809	47-48
48	123	6,645	795	877	48-49
49	173	6,818	622	3,130	49-50
50	142	6,960	480	1,162	50-51
51	74	7,034	406	593	51-52
52	95	7,129	311	3,218	52-53
53	80	7,209	231	939	53-54
54	395	7,440	–	–	–

confirmed the presence of expected fragments after restriction enzyme digestion (Figure 2C). The fragment pool was then cloned into the reporter to generate a plasmid library.

The library and the mini-gene target were transfected alone or co-transfected at an equimolar ratio into HEK293T cells (ATCC, Manassas, Virginia). GFP-positive wells were visualized with fluorescence microscopy and assayed at 48 hr after transfection by flow cytometry to quantify the percentage of GFP-positive cells in each population. Co-transfection of target with reporter PTMs encoding fragments 02 or 07 resulted in the highest expression of GFP, while transfection with the reporter PTMs alone resulted in less than 1% GFP-positive cells (Figure 2D). Interestingly, the reporter with no binding domain (NBD) generated a small fraction of GFP-positive cells when co-transfected with the target. Reporters with a binding domain sequence in forward orientation, demarked "f," were predicted to not improve *trans*-splicing efficiency, and samples 09f and 13f were not above the level of NBD, while 05f was slightly increased (Figure 2D; *BD_05f/09f/13f*). Sequence comparisons between the target and test molecules using ClustalOmega[34,35] and BLASTn[36] did not reveal regions of homology that would be predicted to facilitate targeted *trans*-splicing by canonical mechanisms.

We next validated that GFP signal was resulting from reconstitution of the GFP ORF and not from a post-translational event. We used primers specific to the junction between the 5′ and 3′ portions of GFP to probe cDNA generated from treated cells. A band of expected size was amplified in all samples co-transfected with both the target mini-gene and the reporter molecules, but not when individually transfected (Figure 2E). As *CEP290* is a ubiquitously expressed gene, the test molecules were predicted to also *trans*-splice to endogenous pre-mRNA. To investigate this, we used the same 5′ GFP primer and paired it with a primer binding to *CEP290* exon 27. A band was exclusively detected in cDNA from cells that were transfected with BD_07, and co-transfection reduced the intensity of this band (Figure 2F).

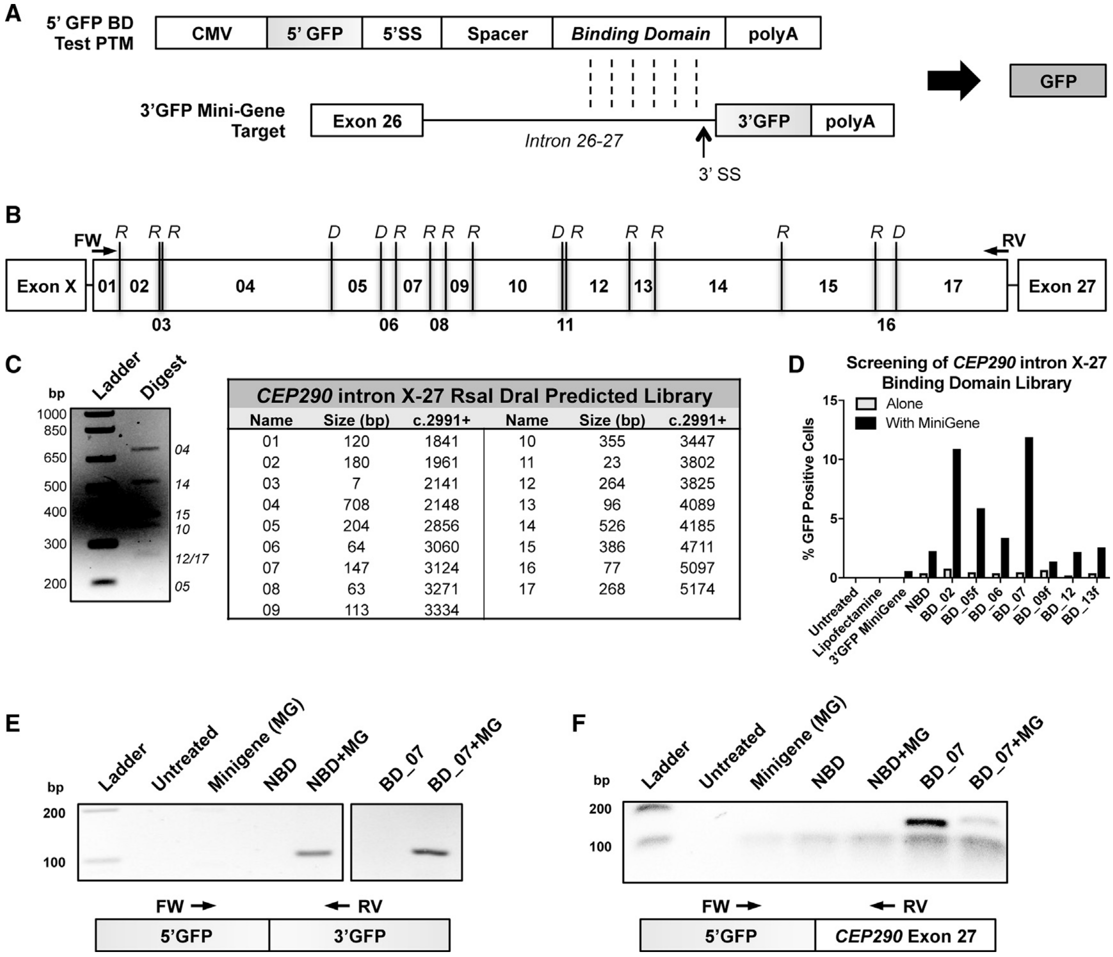

CEP290 intron X-27 RsaI DraI Predicted Library

Name	Size (bp)	c.2991+	Name	Size (bp)	c.2991+
01	120	1841	10	355	3447
02	180	1961	11	23	3802
03	7	2141	12	264	3825
04	708	2148	13	96	4089
05	204	2856	14	526	4185
06	64	3060	15	386	4711
07	147	3124	16	77	5097
08	63	3271	17	268	5174
09	113	3334			

Figure 2. Identification of a Candidate Binding Domain to Target *trans*-Splicing Molecules to *CEP290* Intron X-27

(A) Schematic of *trans*-splicing between a 5′ binding domain (BD) test PTM encoding the 5′ portion of GFP (5′ GFP) and a mini-gene target encoding the 3′ portion of GFP (3′ GFP). Watson-Crick base pairing is indicated by vertical dashed lines. *Trans*-splicing between the two pre-mRNAs results in reconstitution and expression of GFP. (B) Diagram of RsaI (R) and DraI (D) restriction sites within a region of *CEP290* intron X-27. (C) Agarose gel electrophoresis after restriction enzyme digestion of a PCR fragment corresponding to the region described in (B). The fragment was amplified from genomic DNA, digested with restriction enzymes RsaI and DraI, and visualized on a 2% TBE-agarose gel. The fragment library numbers of visible bands of expected sizes are indicated according to the table of predicted fragments. (D) Quantitation by flow cytometry of GFP expression in HEK293T transiently transfected with plasmids encoding a fragment library test PTM (gray bars) or co-transfected with a test PTM and the 3′ GFP target (black bars). Samples with “f” demark forward orientation that is not predicted to confer *trans*-splicing specificity; however, BD_05f did yield a slight improvement to GFP expression over no binding domain (NBD). (E) Agarose gel electrophoresis following RT-PCR using cDNA generated from HEK293T cells transfected with plasmids in (D). Primers were designed to specifically bind to the 5′ or 3′ portions of the GFP coding DNA sequence to validate *trans*-splicing between the 5′ test PTM and the 3′ GFP target pre-mRNAs. Data for the other test PTMs has been removed at the break indicated. Interestingly, untargeted PTM (NBD) also resulted in *trans*-splicing of RNA in agreement with observed GFP expression. MG, mini-gene. (F) Samples from (E) with primers designed to specifically bind to the 5′ portion of GFP or to exon 27 of *Homo sapiens CEP290*. Image has been contrast enhanced to visualize the faint band in lane 7 for co-transfection of BD_07 with target mini-gene (MG).

Editing of Endogenous CEP290 Transcripts in HEK293T by *trans*-Splicing

Since transfection of HEK293T with the screening plasmid resulted in detectable *trans*-splicing of 5′ GFP to endogenous *CEP290*, we then cloned the BD_07 sequence into a PTM encoding the 5′ PCDS of *CEP290*. The presence of a transcriptional terminator on a PTM has been demonstrated to have mixed results, which may be context dependent.[26] Lack of a terminator is expected to retain a pre-mRNA in the nucleus, which could facilitate the *trans*-splicing reaction. To assess this effect in our system, we generated constructs with a bovine growth hormone poly-adenylation signal sequence (PTM_07 PA) or no poly-adenylation sequence (PTM_07 NPA) (Figure 3A).

To elucidate nuanced differences between treatments, we performed qPCR with TaqMan probes on cDNA from HEK293T cells transfected with the 5′ PTMs or a GFP plasmid and isolated RNA at 72 hr post-treatment. Cells treated with PTM_07 PA showed a slight but insignificant reduction of the *CEP290* exon 26-27 junction

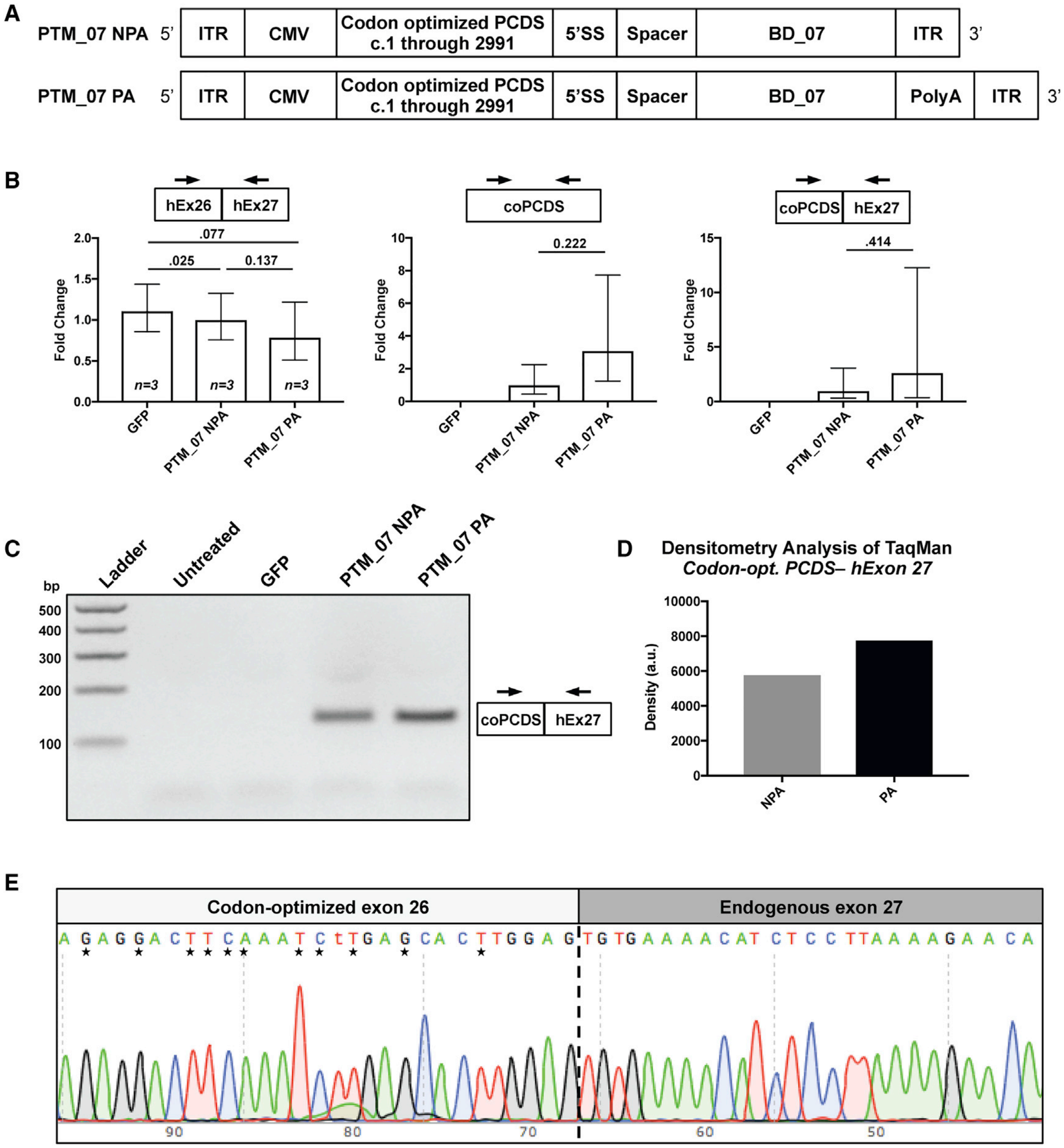

Figure 3. Editing of *CEP290* Transcripts Occurs in HEK293T after Transfection with PTMs

(A) Illustration of adeno-associated virus genome arrangement of a *CEP290* 5′ PTM with binding domain 07 (PTM_07) with either no poly-adenylation signal (NPA) or with a bovine growth hormone poly-adenylation signal (PA). ITR, inverted terminal repeat; CMV, cytomegalovirus promoter; PCDS, partial coding DNA sequence of *CEP290*; 5′ SS, 5′ splice site. (B) qPCR was performed on HEK293T transfected with a plasmid encoding GFP as a transfection control or the plasmids in (A). TaqMan probes were designed to the junctions indicated: *CEP290* exons 26 and 27 (hEx26-hEx27), a region within the 5′ codon optimized partial coding DNA sequence (coPCDS), or the novel junction of 5′ coPCDS and endogenous *Homo sapiens* exon 27 to signify *trans*-splicing (coPCDS-hEx27). Significant variation within replicates for coPCDS-hEx27 was present because the Ct was crossed at 35 cycles with either PTM; however, no amplification was observed through 40 cycles in GFP-treated samples. Samples were standardized to β-2-microglobin and normalized to NPA. Error bars are relative quantity minimum and maximum 95% confidence intervals. (C) Agarose gel electrophoresis of one of the replicate reactions from (B). (D) Densitometry analysis of the bands in (C). (E) Sanger sequencing following TOPO-cloning of the PCR product comprising the coPCDS-hEx27 junction visualized in (C). Nucleotide differences between *Homo sapiens* and codon-optimized *CEP290* are noted by asterisks. The junction between the 5′ coPCDS and endogenous exon 27 is marked by a vertical dashed line.

(Figure 3B, left). We also designed a probe internal to the 5′ codon-optimized sequence that detects overall expression of the 5′ PCDS regardless of *trans*-splicing to endogenous pre-mRNA or of RNA maturation of the PTM transcript without *trans*-splicing. This probe showed a trend toward a 2.5-fold increase in PTM expression for PTM_07 PA when compared to PTM_07 NPA, though it was also

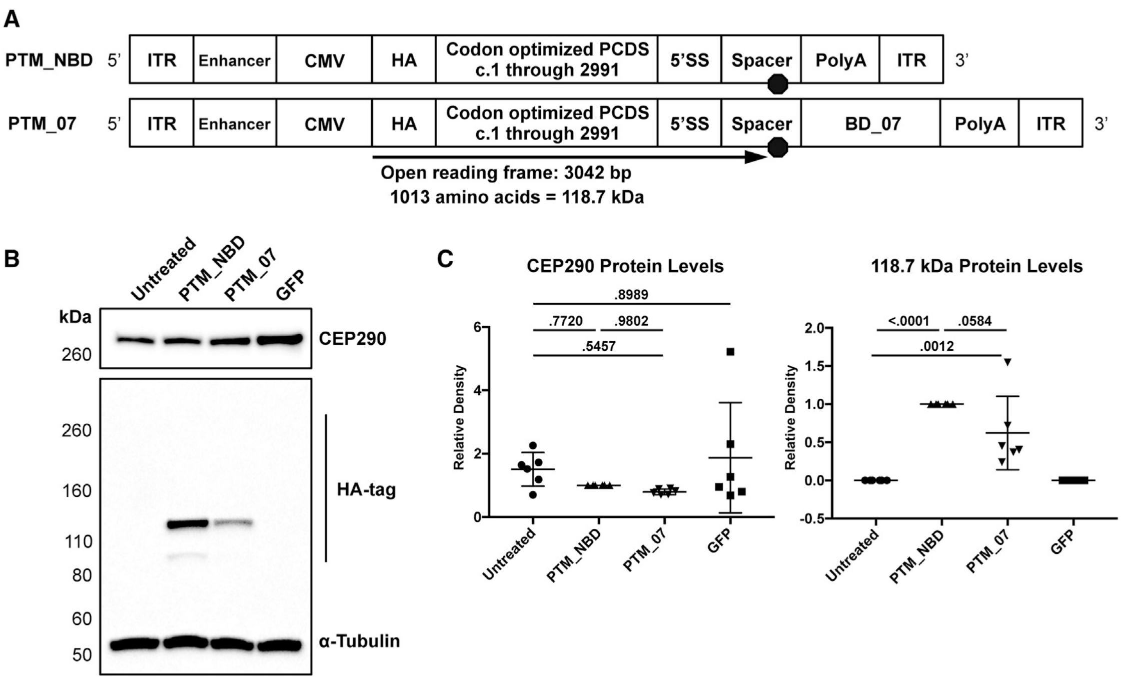

Figure 4. HA-Tagged PTMs Show Significant Maturation following Transfection in HEK293T

(A) *In silico* analysis of both genomes encoding either the 5′ PTM with NBD (PTM_NBD) or with *CEP290* intron X-27 binding domain 07 (PTM_07) indicates an open reading frame (ORF) is present that is predicted to encode a 118.7-kDa peptide if the pre-mRNA matures without *trans*-splicing. (B) Representative western blot images of HA-tag and CEP290 using extracts from HEK293T harvested 72 hr following transfection with the indicated plasmids. HA-tagged CEP290 resulting from *trans*-splicing was expected at ~290 kDa. α-tubulin was used as a loading control. (C) Densitometry quantification of CEP290 and the 118.7-kDa bands. Values per replicate group were standardized to α-tubulin and normalized to PTM_NBD. Error bars are SD; n = 6 independent experiments. $p < 0.05$ was considered significant.

not statistically significant across biological replicates (Figure 3B, center). Similarly, we observed amplification of *trans*-spliced products with treatment by both NPA and PA PTMs with an insignificant 3-fold trend for higher *trans*-splicing in the samples with a poly-adenylation signal (Figure 3B, right). Additionally, we generated similar PTMs lacking a binding domain. We did not detect *trans*-splicing in cells treated with NBD (data not shown). We also validated the *trans*-spliced product by visualizing the PCR product of the *trans*-splicing detection reaction and confirmed a band of the expected size in both PTM_07 NPA- and PA-treated samples, but not untreated or GFP-treated reactions (Figure 3C). Additionally, the band of PTM_07 PA had a slightly higher density than PTM_07 NPA (Figure 3D).

Finally, we further validated the novel splice junction with Sanger sequencing of both the qPCR band and a second PCR amplicon derived from end-point PCR with another primer pair. Analysis of these sequencing reads confirmed the novel junction of codon-optimized exon 26 to endogenous exon 27 with asterisks (*) indicating nucleotide changes designed by codon-optimization (Figure 3E).

Maturation of the PTM Is Detected at High Levels

After observing higher cycle threshold (Ct) values of codon-optimized sequence expression than *trans*-splicing following transfection, we suspected that PTM genomes could be transcribing and generating mature mRNA without *trans*-splicing. We added an amino-terminal HA-tag to PTM_NBD PA and PTM_07 PA to assess peptide production from the PTMs and added the CMV enhancer to further increase expression. *In silico* sequence analysis of the 5′ PTM revealed an ORF that could generate a predicted peptide of 118.7 kDa (Figure 4A). To assess if this peptide was indeed present, we performed western blot analysis of protein extracts from HEK293T transfected with the HA-tagged PTMs. This revealed an HA-tagged protein between 110 kDa and 160 kDa with a minor band between 80 and 110 kDa, while no HA-tagged protein was detected above 260 kDa (Figure 4B). A 290 kDa band would be expected for *trans*-splicing to endogenous *CEP290.* There was neither significant change to the levels of CEP290 protein following transfection nor a statistically significant difference in levels of the 118.7 kDa peptide between PTM_NBD and PTM_07 (Figure 4C).

The addition of a downstream intronic splice enhancer (DISE) derived from rat *FGFR2* has been demonstrated to promote inclusion of exons and PTMs.[37,38] We inserted the corresponding sequence into the spacer region of PTM_07 and performed transfections in HEK293T. We observed no significant differences in RNA or protein levels between treatment with PTM_07 or PTM_07 *FGFR2* DISE (data not shown).

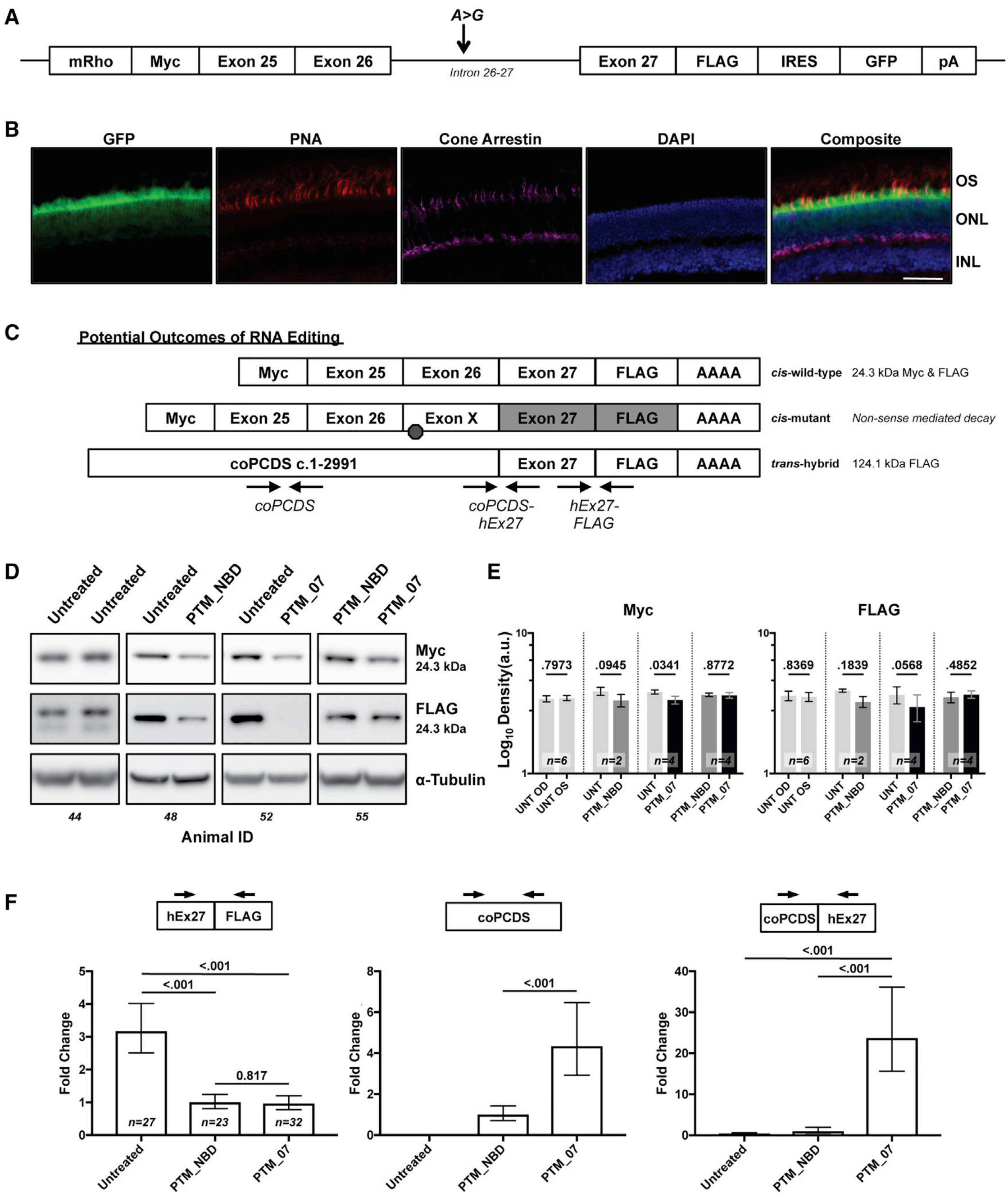

Figure 5. Editing of Mini-*CEP290* Transcripts Occurs *In Vivo* following Sub-retinal Injection of 7m8AAV-5′ PTMs

(A) Diagram of the *CEP290* intron 26 mini-gene. The mini-gene is driven by the murine *Rhodopsin* promoter (mRho). *CEP290* exons 25 and 26 are joined and followed by the complete intron 26-27 and exon 27. An A > G mutation corresponding to c.2991+1655 was included to assess exon X splicing in the model. An amino-terminal Myc and a carboxy-terminal FLAG tag were added to flank the mini-gene. Additionally, an internal ribosomal entry site (IRES) was added to drive expression of EGFP and is terminated by a bovine growth hormone poly-adenylation signal sequence (pA). (B) Immunofluorescence staining of a retinal cross-section from a mini-*CEP290* mouse. OS, outer segment. ONL, outer nuclear layer. INL, inner nuclear layer. Scale bar is 50 μm. (C) Illustration of potential splicing outcomes within the mini-*CEP290* mouse. Canonical *cis*-splicing would result in a predicted 24.3-kDa peptide. Alternative *cis*-splicing with exon X would yield a predicted non-sense media decay transcript encoding a truncated peptide with Myc at 17.1 kDa and no FLAG translation. *Trans*-splicing with a 5′ PTM would replace the amino-terminal Myc and generate a 124.1-kDa peptide with a FLAG tag. Arrow pairs indicate locations of PCR primers used to detect specific splicing events. (D) Representative western blot images of Myc and FLAG at 24.3 kDa showing OD and OS lanes per animal for each contralateral treatment cohort. (E) Densitometry quantification showing the mean $\log_{10}$ values of each contralateral treatment cohort. Samples were standardized to α-tubulin. Error bars are SEM. Sample sizes as indicated. Individual animal data is available in Figure S1A. (F) qPCR from cDNA generated by RNA extracts

(legend continued on next page)

Evaluating *trans*-Splicing in a Murine Mini-gene Model of hCEP290 Intron 26-27

To demonstrate *in vivo* proof-of-concept pre-mRNA editing of this region of *CEP290*, we utilized an engineered mouse model.[39] This model contains an LCA10 mini-gene driven by the murine sequence immediately upstream to the *Rhodopsin* gene and encoding the genomic region of *Homo sapiens CEP290* exons 25, 26, intron 26 carrying the IVS26 mutation, and exon 27. The promoter selection limits expression of the mini-gene to rod photoreceptor cells. A detected *trans*-splicing event can thus be presumed to be the product of activity in photoreceptors, even in samples that may contain material from other cell types. Additionally, a 5′ Myc tag and a 3′ 3×FLAG tag are in-frame with the *CEP290* coding sequences. The cassette is followed by an internal ribosomal entry site and enhanced GFP coding sequence (Figure 5A). Expression of GFP was confirmed to be restricted to photoreceptors in the outer nuclear layer and the transition zone between the outer nuclear layer and outer segments (Figure 5B). In this model (which was not designed to manifest disease), normal *cis*-spliced products are expected to generate a 24.3-kDa peptide, while a *trans*-spliced product would generate a novel 124.1 kDa FLAG band (Figure 5C). Additionally, *trans*-splicing would be predicted to reduce the amount of 24.3 kDa Myc and FLAG protein.

To investigate these changes, we packaged the proviral PTM genomes in the AAV7m8 capsid, which has been shown to be effective for efficient transduction *in vivo* in mouse and primate retinas as well as *in vitro* for several cell lines.[40] We then performed sub-retinal injections of control and experimental AAVs at post-natal day 15 and harvested whole eyes for either RNA or protein analysis 30 days post-injection. Treatment with the control AAV7m8-PTM_NBD and the experimental AAV7m8-PTM_07 trended toward reduction of Myc- and FLAG-tagged proteins at 24 kDa, though only Myc was statistically significant (Figures 5D, 5E, and S1A). FLAG detection of a novel 124.1 kDa band, an indicator of *trans*-splicing activity, was not observed in any sample (data not shown). However, we also observed baseline variability between the untreated eyes of animals for Myc and FLAG protein levels in several untreated mice (Figure S1A, cohort A).

To assess if we were observing an effect of RNA editing and not exclusively an artifact of AAV injection, we generated cDNA from additional mice and evaluated transcript levels using TaqMan probes specific to (1) the *Homo sapiens* exon 27-FLAG junction to gauge expression of all mini-gene transcripts including *cis*-spliced or *trans*-spliced, (2) an internal sequence of the codon-optimized PCDS to gauge expression of all PTM-generated transcripts including *cis*-spliced or *trans*-spliced, or (3) the novel junction specifically indicating *trans*-splicing of the codon-optimized PCDS to *Homo sapiens* exon 27. Analysis of samples by biological groups revealed that injection of either PTM_NBD or PTM_07 reduced overall expression of the mini-gene by approximately 75% (Figure 5F, left). Total levels of PTM_07 were detected as 4-fold higher than PTM_NBD, while no detection was observed in untreated samples (Figure 5F, center).

Despite the higher overall levels of PCDS expression observed following treatment with PTM_07, *trans*-splicing of the codon-optimized 5′ PCDS was detected with either treatment, but, importantly, levels were 25-fold higher with PTM_07 (Figure 5F, right). Individual data grouped by contralateral eye treatments demonstrate variability between mice but show consistently that *trans*-spliced products are detected at higher levels after treatment with PTM_07 relative to PTM_NBD, and in 5 out of 27 untreated eyes, some level of *trans*-splicing was detected but at amounts resembling PTM_NBD (Figure S1B, bottom). One animal that was injected with PTM_07 had no visible bleb after injection. Interestingly, in this sample, the level of codon-optimized PCDS was reduced, and *trans*-splicing was not detected (Figure S1B, animal 26). This sample provided evidence that successful sub-retinal injection of AAV7m8 PTM vectors is required to mediate *trans*-splicing activity to a photoreceptor-specific target.

DISCUSSION

Gene augmentation and editing strategies for targets with a coding sequence greater than 4,000 nt face many challenges. The size of delivery vectors imposes a restriction on which targets can be utilized in canonical gene augmentation. In this study, we have shown that spliceosome-mediated pre-mRNA *trans*-splicing can be utilized to edit transcripts of *CEP290*, which causes severe early-onset blindness (LCA10), which can also be accompanied by systemic disease. We created an analysis paradigm that can be applied to any gene target to maximize potential benefit. Further refinements to the components of the PTMs will allow for optimization of the analysis parameters. With regards to *CEP290*, we could target a PTM downstream of cryptic exon X. This approach could ultimately correct at least one allele in nearly 70% of LCA10 patients and approximately 50% of all reported loci with mutations within *CEP290*.

Many studies have also shown that 3′ PTMs can replace the 3′ region of genes. By applying our analysis paradigm in the reverse direction, one can also calculate a maximal 3′ PCDS size (Table 1). Through the two approaches, nearly 90% of reported *CEP290* mutation loci can hypothetically be targeted.

Our low-throughput screen identified two candidate binding domains that could reconstitute GFP. High-throughput screens have been utilized in a variety of other studies by applying techniques including sonication to generate robust libraries.[23,24,29,31,41] Regardless of

from whole eyes of mini-*CEP290* mice. TaqMan probes were designed to the junctions of *Homo sapiens* exon 27 and FLAG to detect total expression of the mini-gene (left) to a region within the PCDS to detect total expression of the PTM (center) or to the novel junction of codon-optimized *CEP290* PCDS and *Homo sapiens CEP290* exon 27 (right). Values from treatment-matched samples were averaged as biological groups, standardized to murine β-2-microglobin and normalized to PTM_NBD. $p < 0.05$ was considered significant. Error bars are relative quantity minimum and maximum 95% confidence intervals. Sample sizes as indicated. Individual animal data is available in Figure S1B.

screening method, efficiency of *trans*-splicing continues to be the rate-limiting step for efficacy. Indeed, in another study, repair of *Rho* showed *in vivo trans*-splicing and protein production after sub-retinal injection but did not demonstrate phenotypic rescue.[17] The ideal binding domain length, nucleotide composition, and other parameters have yet to be ascertained. Ongoing studies will identify what properties influence binding-domain characteristics and *trans*-splicing efficiency. There may be additional features that affect efficiency of *trans*-splicing, such as the efficiency of splicing in the native molecule.[42]

Our negative control of the untargeted PTM with NBD unexpectedly did reconstitute the ORF in our GFP screen. Additionally, three of the GFP-positive library clones had binding-domain fragments in forward orientation, which were expected to not confer specificity of the PTM (05f, 09f, 13f). Since the levels of the forward binding-domain clones were not significantly higher than the no-binding-domain sample, the observation may be the result of non-specific, random *trans*-splicing resulting from an abundance of transcription template following transfection. Transcripts resulting from expression of transfected plasmids may be compartmentalized such that there is an increase in the local concentrations of the two molecules sufficient for stochastic *trans*-splicing. Further studies including *in situ* hybridization will help identify the cause of this untargeted *trans*-splicing.

Regarding the transcriptional terminator, our findings suggest that the poly-adenylation signal sequence increased PTM expression, and this correlated with higher *trans*-splicing, albeit not statistically significant. The basis for removal of the transcriptional terminator was to prevent maturation of the pre-mRNA and promote nuclear retention since the molecule would lack the elements required for nuclear export. Increasing heterogeneous nuclear ribonucleoprotein (hnRNP) binding sites may be another option for preventing export from nucleus. However, we cannot rule out other variables such as RNA structure that may be independent of the poly-adenylation tail and a result of changes to the overall PTM genome. Indeed, PTMs targeting murine *dystrophin* were improved with an intron splice enhancer sequence.[38] Addition of this particular enhancer did not improve *trans*-splicing in our system, but there may be alternative sequences that can improve spliceosome recruitment in this context. A thorough analysis of additional transcriptional terminators and other intron binding elements on *trans*-splicing molecules may help reveal copy number, RNA structure, and other context-dependent effects that can contribute to future optimizations.

Specificity of the *trans*-splicing reaction to endogenous transcript conferred by the binding domain was confirmed by comparing transfection of BD_07 alone or BD_07 co-transfected with the mini-gene. Reduction in the *trans*-spliced 5′ GFP—exon 27 product when co-transfected reflects specificity of the binding domain since overexpression of the target mini-gene would create additional target sites for the PTM transcripts.

By probing for the HA-tag on our 5′ codon-optimized *CEP290* PTM, we discovered that translation of the PTM occurred with a peptide at the size predicted by an ORF present in the PTM. This ORF is only present if the 5′ splice site does not prevent maturation of the pre-mRNA. With BD_07 present, *in silico* analysis revealed multiple putative stop codons for the ORF. Mutation of the PTM to remove these putative stop codons would require changes to not only the spacer but also within the binding domain, which may reduce specificity and binding efficiency. Alternative transcriptional terminators and additional nuclear retention elements may be required to reduce this *cis*-splicing and increase the retention of PTM pre-mRNA, which, per the earlier poly-adenylation signal work, could increase *trans*-splicing availability.

Mini-gene systems have been used to dissect alternative splicing in a variety of models.[43] When the *Homo sapiens CEP290* exon 26, intron 26 with IVS26, and exon 27 region was targeted for specific insertion into the homologous region in a mouse, an mRNA species was identified that retained a second cryptic exon, dubbed exon Y, which is not observed in any human cDNA samples.[44] This finding suggested that splicing in the mouse may not accurately recapitulate human splicing as a model of IVS26.

We thus investigated the utility of a tagged mini-gene containing only the region of interest when expressed in mouse photoreceptors. Variability in both baseline levels of mini-gene and in expression of transduced 5′ PTMs was observed between mice and is potentially due to biological and/or injection variability resulting in dose variations. However, dose variation alone would not account for the 25-fold overall difference seen for PTM_07 *trans*-splicing as a group or the consistently higher levels of *trans*-splicing in individual animals treated with PTM_07 compared to the contralateral eye treated with PTM_NBD. With Ct values after 35 cycles, the PTM_NBD signal is likely the result of rare un-targeted *trans*-splicing events. These same events were observed in the initial screen when using the 5′ GFP with NBD. Additionally, five untreated samples from cohort C had slight amplification with a mean value significantly less than that of PTM_NBD, which may indicate that the primer-probe set may not be perfectly specific.

We validated the PCR products to ensure the observed signal was not a result of secondary sources such as primer dimers or non-specific amplification within the sample. Non-specific *trans*-splicing may be resolved by improved engineering of the *trans*-splicing molecules and will be important to consider in future safety studies of *trans*-splicing.

Efficiency of *trans*-splicing molecules may also be context specific. Our mouse model maintained a higher genomic copy number of the mini-gene, which may be contributing to increased pre-mRNA expression and *trans*-splicing. It will be important to evaluate *trans*-splicing in additional models including patient-derived samples. Regarding LCA10, patient-derived induced pluripotent stem cells have been shown to have lower levels of *CEP290* transcripts.[45]

Evaluating *trans*-splicing in these models, including nuclear fractionation to detect pre-mRNA, will help reveal if pre-mRNA is present in sufficient quantities to facilitate *trans*-splicing.

Collectively, we have demonstrated that 5′ pre-mRNA *trans*-splicing can be targeted to edit *CEP290* transcripts. This establishes an approach that can be further optimized to correct a broad range of patient mutations in the region 5′ to and including IVS26. Contrary to other methods of treatment, this approach creates the opportunity to correct both alleles. The approach can ultimately be expanded to other monogenic ocular and extra-ocular disease-associated genes that are too large to fit in AAV.

MATERIALS AND METHODS

In Silico Analysis

Nucleic acid sequences were analyzed, and Sanger sequencing alignments were performed with SnapGene software (GSL Biotech, Chicago, IL). Analysis of real-time PCR results was performed with ExpressionSuite Software v1.1 (Applied Biosystems). Graphs and statistical calculations were generated with GraphPad Prism v7 (GraphPad Software, La Jolla, CA). Densitometry analysis of immunoblot images was performed with ImageJ software (https://fiji.sc). Computation of densitometry values was performed using Microsoft Excel.

Statistics

Densitometry measurements of western blots were standardized to sample-matched α-tubulin values. For HA-tagged PTM experiments (Figure 4), six independent transfections were performed, and protein lysates were evaluated by immunoblotting. Densitometry values were normalized to PTM_NBD-treated lanes. Adjusted densitometry values of peptides of interest were compared in Graphpad Prism software using one-way ANOVA followed by Tukey's multiple comparison test. A p value less than 0.05 was considered statistically significant.

Ct values of TaqMan PCR were standardized to the β-2-microglobin control gene. Samples with matching treatments were considered biological groups, and mean ΔCt values were computed per group. All reactions were performed in technical triplicates for each biological replicate and processed with the ΔΔCt method. Values were normalized to PTM_NPA (Figure 3) or PTM_NBD (Figure 5). The means of biological groups were compared via Student's t test with confidence intervals calculated at 95%. A p value less than 0.05 was considered statistically significant.

A post-hoc power analysis was conducted for the *trans*-splicing Ct values of the *in vivo* studies with a two-sample one-sided test. With an alpha of 0.05, the power was greater than 0.999 when comparing cohorts and the study included more than the minimal number of samples for significance.

Culture Conditions and Transfection of HEK293T

HEK293Ts were maintained in DMEM with GlutaMax (Thermo Fisher Scientific, Waltham, MA) supplemented with 10% (v/v) fetal bovine serum (GE Healthcare, Little Chalfont, UK) and 100 U/mL Pen Strep (Thermo Fisher Scientific). Dissociation of cells was achieved by washing with Dulbecco's PBS (DPBS; Corning, Corning, NY) and incubation with Trypsin-EDTA 0.05% (v/v) (Thermo Fisher Scientific).

All transfections were performed with Lipofectamine LTX (Thermo Fisher Scientific) per manufacturer's instructions at a 3:1 ratio of reagent to plasmid. For binding domain screening, 5×10^4 cells were plated in 24-well cluster plates. 1 μg of library clone plasmid was transfected either alone or with equimolar target plasmid along with an equal volume per weight of PLUS reagent. For protein extracts, 3×10^5 cells were plated in 6-well cluster plates and transfected with 2.5 μg plasmid with an equal volume per weight of PLUS reagent. At harvest, 10% of dissociated cells were separated for RNA and 90% for protein.

cDNA Preparation and RT-qPCR

RNA was isolated from whole mouse eyes following euthanasia and enucleation. Eyes were stored in 500 μL of RNA*later* (Thermo Fisher Scientific) and homogenized by electric pestle in 350 μL Buffer RLT Plus. RNA was isolated from cell pellets or homogenized eyes with the RNeasy Plus Kit (QIAGEN, Germantown, MD) per manufacturer's instructions following homogenization through QIAshredder columns (QIAGEN). Total RNA concentration was quantified on NanoDrop 8000 spectrophotometer (Thermo Fisher Scientific). Reverse transcription was performed with Superscript III 1st Strand Synthesis (Thermo Fisher Scientific). qPCR was performed with TaqMan Fast Universal PCR Master Mix (Applied Biosystems, Foster City, CA) with cycling performed in an Applied Biosystems 7500 Fast Real-Time PCR system. Stock primer probes included human and murine beta-2-microglobin (Thermo Fisher Scientific). Custom primer-probes were ordered from Thermo Fisher Scientific for codon-optimized PCDS internal FW 5′-ACC AAG GAA ATC AAT AAG CTG GAA CT-3′, RV 5′-GAT CAT TGT TTT GGG TTC CAG TCC TA-3′, probe 5′-CAG GGC TTC GTT CTC ATC G-3′; or CEP290 exon 27-FLAG FW 5′-ACT ATT GAA CAA GCC TGG GAA CAG-3′, RV 5′-TCA TCC TTG TAA TCG ATG TCA TGA TCT TT-3′, probe 5′-TTT GTA GTC CCC TAA TTT AG-3′. The codon-optimized exon 26 to endogenous exon 27 for detection of *trans*-splicing was ordered from Integrated DNA Technologies: FW 5′-GTT GGT CCA GAG GAC TTC AAA-3′, RV 5′-TAT TGA ACA AGC CTG GGA ACA-3′, probe 5′-TCT TGA GCA CTT GGA GTG TGA AA-3′.

Protein Extraction

Protein extracts were isolated from either cell pellets or whole eyes by lysis buffer consisting of Radioimmunoprecipitation assay (RIPA) Buffer (Cell Signaling Technologies, Danvers, MA) supplemented with cOmplete proteasome inhibitor (Roche, Basel, Switzerland). Following euthanasia and enucleation, whole eyes were homogenized in 250 μL of RIPA Buffer by electric pestle. Cell pellets were resuspended in RIPA at a 1:1 volume to the cell pellet. Cell or eye lysis solutions were vortexed for 30 s, rotated end-over-end for 30 min at

4°C, vortexed for 30 s, centrifuged at 21,000 × *g* for 20 min at 4°C, and protein-containing supernatant was harvested. Quantification of protein concentration was performed with Micro BCA Protein Assay Kit (Thermo Fisher Scientific).

Western Blotting

Protein samples were separated via the NuPage Electrophoresis System (Thermo Fisher Scientific) per manufacturer's instructions. Samples were heated at 70°C for 10 min and loaded to NuPAGE 3%–8% Tris-Acetate (Figure 4) or NuPage 4%–12% Bis-Tris (Figures 5 and S1) Protein Gels (Thermo Fisher Scientific). Novex Sharp Prestained Protein Standards (Thermo Fisher Scientific) was used to mark protein band sizes. Separated proteins were then transferred in an XCell II Blot Module (Thermo Fisher Scientific) to Immun-Blot polyvinylidene difluoride membranes for Protein Blotting (Bio-Rad, Hercules, CA) at 30 V for 1 hr. After transfer, membranes were incubated in Tris-buffered saline with 0.1% v/v Tween 20 (Bio-Rad) supplemented with 5% (w/v) non-fat dry milk (LabScientific, Highlands, NJ) for 1 hr at room temperature. Primary antibodies and respective dilutions included: rabbit polyclonal anti-CEP290 (Abcam, Cambridge, MA) 1:1,000; mouse monoclonal antibody (mAb) anti-tubulin DM1A (Cell Signaling Technologies) 1:1,000; mouse mAb β-actin 8H10D10 (Cell Signaling Technologies) 1:2,000; rabbit mAb HA-Tag C29F4 (Cell Signaling Technologies) 1:500; mouse mAb Myc-Tag 9B11 (Cell Signaling Technologies) 1:500; mouse mAb FLAG M2 (Sigma-Aldrich, St. Louis, MO). Primary antibodies were diluted in Tris-buffered saline with 0.1% v/v Tween-20 (TBST)-5% milk and incubated with respective membrane portions at 4°C overnight. Membranes were washed 3 × 15 min in TBST. Secondary antibodies anti-mouse horseradish peroxidase (HRP) enhanced chemiluminescence (ECL) (GE Healthcare) 1:10,000 or anti-rabbit HRP ECL (GE Healthcare) 1:10,000 were diluted in TBST-5% milk and incubated with membranes for 1 hr at room temperature. Membranes were washed then incubated with ECL2 (Thermo Fisher Scientific) per manufacturer's instructions for 5 min. Membranes were imaged with an Amersham Imager 600 (GE Healthcare) with chemiluminescence settings.

Generation of PTM Binding Domain Reporter Library

Aequorea coerulescens GFP (*AcGFP*) 5′ binding domain reporter plasmid was a gift from Lloyd Mitchell (Retrotherapy, Bethesda, MD). The 5′ GFP reporter consists of a 5′ *GFP* portion encoding Met1 through Glu112 and is followed by a canonical GTAAG 5′ splice site, a 20-nt spacer sequence, and a multiple cloning site. The reporter vector was digested with the blunt cutting endonuclease EcoRV and dephosphorylated with recombinant Shrimp Alkaline Phosphatase (New England Biolabs, Ipswich, MA). The treated reporter was separated by size-exclusion on Tris-borate-EDTA (TBE)-agarose gel electrophoresis and a band of expected size was excised from the gel and column purified with NucleoSpin Gel and PCR Cleanup Kit (Macherey-Nagel, Bethlehem, PA).

A 4,000-nt target region of *CEP290* intron X-27 was amplified from genomic DNA of a healthy donor by PCR using Q5 Hot Start High-Fidelity 2× Master Mix (New England Biolabs). Primers were designed to be offset from the splice sites of exon X and exon 27: *I26 FW 4kb* 5′-CCA GGA TGG TGT CGA TCT CC-3′ and *I26 RV 4kb* 5′-TCT TCT AAT TCC GGC CAC CA-3′. Reactions were separated by size exclusion on TBE-agarose gel by electrophoresis and a band of expected size was excised from the gel. Gel pieces were then column purified using NucleoSpin. The purified fragment was digested with the blunt-end-generating restriction enzymes DraI and RsaI in CutSmart Buffer (New England Biolabs) at 37°C for 2 hr then column purified with NucleoSpin.

A heterogeneous pool of the digested target region was ligated to the blunt-digested reporter using T4 Ligase (New England Biolabs) with an overnight incubation at 16°C. The ligation reaction was transformed into Top10 competent *E. coli* (Thermo Fisher Scientific) per manufacturer's instructions. Transformed cells were spread on LB-Agar plates containing 100 μg/mL ampicillin. Plates were inverted and incubated overnight at 37°C. Clones were screened by colony PCR to identify insert size with the primers *acGFP FW2* 5′-ATC ACA TGA AGC AGC ACG AC-3′ and *V5Tag-RVSeq* 5′-GGA GAG GGT TAG GGA TAG GC-3′. Cultures of unique clones were grown and later Sanger sequence verified to generate the binding domain library.

Generation of Reporter Target Mini-gene

A 3′ *AcGFP trans*-splicing target mini-gene was designed encoding human *CEP290* exons 25 and 26, intron 26, and the 3′ portion of *AcGFP* to complete the coding DNA sequence of the 5′ GFP reporter. This mini-gene was generated by in-fusion cloning (Macherey-Nagel) to create a proviral lenti-plasmid with CMV immediate-early promoter, CMV enhancer, Kozak consensus sequence, ATG start codon, *CEP290* exon 26 and intron 26, the 3′ portion of GFP, a STOP codon, and the bovine growth hormone poly-adenylation signal sequence.

To generate a positive control for full-length *GFP*, the 5′ *GFP* and 3′ *GFP* sequences were assembled via in-fusion cloning using the respective 5′ and 3′ PTMs as templates. The fragments were inserted into an expression plasmid driven by the CMV immediate-early enhancer and promoter.

Screening of Binding Domains

Library screening was performed by co-transfection of PTM Target 3′ *GFP* and reporter PTM binding domain library clones into HEK293T at an equal molar ratio. At 48 hr post-transfection, cells were dissociated, and the fraction of GFP-positive cells was determined by flow cytometry using BD Accuri C6 (BD Biosciences, San Jose, CA). Samples were gated using a GFP plasmid transfection in which 99.9% of cells were GFP positive (data not shown).

Confirmation of *trans*-spliced RNA was evaluated by reverse transcription followed by PCR using the primers *GFP FW1* 5′-TTC GAG GAT GAC GGC AAC TA-3′ and *GFP RVseq* 5′-GCC ATC CTC CTT GAA ATC GG-3′. Library clones that showed the highest GFP were assessed for *trans*-splicing to endogenous *CEP290* with

primers *GFP FW1* 5′-TTC GAG GAT GAC GGC AAC TA-3′ and *hCEP290 E27 RVseq* 5′-CCC AGG CTT GTT CAA TAG TGT-3′.

Generation of CEP290 5′ PTM

PCR was utilized to amplify the 5′ PCDS from a codon-optimized human *CEP290* coding DNA sequence (CDS) (DNA 2.0 now ATUM, Newark, CA) and a 5′ splice site and the first 20 nucleic acids of *hCEP290* intron 26 were added via the oligonucleotide primers. This fragment was cloned into the ScaI site of the AAV proviral vector p1107 (Bennett Lab, Philadelphia, PA) to generate PTM_NBD. PTM_07 was generated by amplifying the binding domain 07 sequence from the 5′ *GFP* clone BD15 and inserting this fragment into the EcoRV site of PTM_NBD. All constructs were validated by Sanger sequencing.

Immunohistochemistry

Whole eyes were isolated by enucleation of euthanized animals. Eyes were fixed in 4% paraformaldehyde for 2 hr on ice and then cryoprotected with 30% (w/v) sucrose in DPBS. Eyes were then embedded in Tissue-Tek optical cutting temperature cryopreservation media (Sakura Finetek USA, Torrance, CA) and frozen at −80°C. Embedded eyes were sectioned on a Leica CM1850 cryostat (Leica Biosystems, Buffalo Grove, IL) at 12 μM thickness and transferred to charged glass microscope slides (Globe Scientific, Paramus, NJ). Tissue slices were then incubated for 1 hr at room temperature in blocking buffer comprised of 10% (v/v) goat serum (Sigma-Aldrich) and 0.5% (v/v) Triton X-100 (Roche) in DPBS (Corning). Primary antibodies of Rhodamine labeled Peanut Agglutinin (Vector Laboratories, Burlingame, California) 1:100 and Cone Arrestin (Abcam) 1:100 were diluted in blocking buffer and applied to samples in a humidified chamber and incubated for 4 hr at room temperature. Slides were then washed three times for 5 min per wash in phosphate buffered saline with 0.5% v/v Triton X-100 (PBST) comprised of 0.5% v/v Triton X-100 in DPBS. Secondary antibody goat anti-rabbit Cy5 (SeraCare, Milford, MA) was diluted in blocking buffer 1:100 and applied to cells for 1 hr at room temperature followed by three washes in PBST and a final wash in DPBS. Fluoromount-G mounting media (Thermo Fisher Scientific) supplemented with DAPI was added to slides prior to the addition of No. 1 1/2 cover glass (Corning) and sealed with nail polish (Electron Microscopy Sciences). 10 Z-slices of 1 μM depth were obtained with a FLUOVIEW FV1000 confocal laser scanning microscope with a PLAPON 60× Oil NA:1.42 objective (Olympus, Center Valley, PA). Images were excited with lasers at wavelengths 405, 488, and 559 or 405 and 635 nm. z stacks between images were aligned with FIJI software using Split Channels, then the 635 nm channel was combined with the other three channels using Merge Channels. The composite image was then flattened using Z Project with Standard Deviation. The final image was rotated and cropped for display orientation.

AAV Vector Production

Vectors were produced by the Center for Advanced Retinal and Ophthalmic Therapeutics Retinal Research Vector Core (Philadelphia, PA). Viral lots were AAV7m8 p628 9.75e12 vector genomes (vg)/mL CT-117, AAV7m8 CMVie coC290 PTM NBD 8.86e12 vg/mL CT-332, and AAV7m8 CMVie coC290 PTM BD15 7.45e12 vg/mL CT-333.

In Vivo Studies

Animals were housed and maintained in accordance with the Association for Research in Vision and Ophthalmology's Statement for the Use of Animals in Ophthalmic and Visual Research and Institutional Animal Care and Usage Committee approval (IACUC #805890). The mini-CEP290 mouse line was generated by pronuclear injection of B6AF1 zygotes with a *CEP290* intron 26 mini-gene driven by the 3.8 kb murine rhodopsin upstream sequence (see Figure 5A). The mini-CEP290 mouse line #406 was generated and maintained by brother-sister matings. Subretinal injections were performed as previously described.[46] Each retina received 8×10^9 vector genomes in a volume of 1 μL PBS supplemented with Pluronic F-68 NF Prill Poloxamer 188.

SUPPLEMENTAL INFORMATION

Supplemental Information includes one figure and can be found with this article online at https://doi.org/10.1016/j.omtn.2018.05.014.

AUTHOR CONTRIBUTIONS

Conceptualization, S.J.D., K.J.F., L.G.M., J.B.; Methodology S.J.D., D.S.M., L.G.M., J.B.; Validation, Formal Analysis, Data Curation, Visualization, and Writing – Original Draft, S.J.D.; Design, Generation and Characterization of Mouse Model: J.B., J.L.B.; Investigation, S.J.D., D.S.M.; Resources, S.J.D., D.S.M., J.L.B., K.J.F., J.B.; Supervision and Funding Acquisition, J.B. All authors reviewed and edited the final manuscript.

CONFLICTS OF INTEREST

S.J.D., J.L.B., L.G.M., and J.B. are co-inventors on patent PCT/US16/62941 relevant to the topic of this article. This patent has been licensed to Limelight Bio., Inc. (Philadelphia, PA), of which J.B. is a co-founder.

ACKNOWLEDGMENTS

We are grateful to Jennifer Pham for maintaining the mini-*CEP290* mouse line, Mary Klein for assistance with GraphPad Prism and other graphics, Zhangyong Wei for expert histologic assistance, and the CAROT Research Vector Core (University of Pennsylvania) and Dr. Shangzhen Zhou, director, for production of AAV. This work was supported by a sponsored research agreement from Limelight Bio, the Foundation Fighting Blindness-sponsored CHOP-Penn Pediatric Center for Retinal Degeneration, the NIH, National Eye Institute grants 5R24EY019861 and T32 EY007035-38, the Brenda and Matthew Shapiro Stewardship, the Robert and Susan Heidenberg Investigative Research Fund for Ocular Gene Therapy, Research to Prevent Blindness, the Paul and Evanina Mackall Foundation Trust, the Center for Advanced Retinal and Ocular Therapeutics, and the F. M. Kirby Foundation.

REFERENCES

1. Koenekoop, R.K. (2004). An overview of Leber congenital amaurosis: a model to understand human retinal development. Surv. Ophthalmol. *49*, 379–398.
2. Simonelli, F., Maguire, A.M., Testa, F., Pierce, E.A., Mingozzi, F., Bennicelli, J.L., Rossi, S., Marshall, K., Banfi, S., Surace, E.M., et al. (2010). Gene therapy for Leber's congenital amaurosis is safe and effective through 1.5 years after vector administration. Mol. Ther. *18*, 643–650.
3. Bennett, J., Wellman, J., Marshall, K.A., McCague, S., Ashtari, M., DiStefano-Pappas, J., Elci, O.U., Chung, D.C., Sun, J., Wright, J.F., et al. (2016). Safety and durability of effect of contralateral-eye administration of AAV2 gene therapy in patients with childhood-onset blindness caused by RPE65 mutations: a follow-on phase 1 trial. Lancet *388*, 661–672.
4. Russell, S., Bennett, J., Wellman, J.A., Chung, D.C., Yu, Z.F., Tillman, A., Wittes, J., Pappas, J., Elci, O., McCague, S., et al. (2017). Efficacy and safety of voretigene neparvovec (AAV2-hRPE65v2) in patients with RPE65-mediated inherited retinal dystrophy: a randomised, controlled, open-label, phase 3 trial. Lancet *390*, 849–860.
5. den Hollander, A.I., Black, A., Bennett, J., and Cremers, F.P.M. (2010). Lighting a candle in the dark: advances in genetics and gene therapy of recessive retinal dystrophies. J. Clin. Invest. *120*, 3042–3053.
6. Parfitt, D.A., Lane, A., Ramsden, C.M., Carr, A.J., Munro, P.M., Jovanovic, K., Schwarz, N., Kanuga, N., Muthiah, M.N., Hull, S., et al. (2016). Identification and Correction of Mechanisms Underlying Inherited Blindness in Human iPSC-Derived Optic Cups. Cell Stem Cell *18*, 769–781.
7. Burnight, E.R., Wiley, L.A., Drack, A.V., Braun, T.A., Anfinson, K.R., Kaalberg, E.E., Halder, J.A., Affatigato, L.M., Mullins, R.F., Stone, E.M., and Tucker, B.A. (2014). CEP290 gene transfer rescues Leber congenital amaurosis cellular phenotype. Gene Ther. *21*, 662–672.
8. Ruan, G.X., Barry, E., Yu, D., Lukason, M., Cheng, S.H., and Scaria, A. (2017). CRISPR/Cas9-Mediated Genome Editing as a Therapeutic Approach for Leber Congenital Amaurosis 10. Mol. Ther. *25*, 331–341.
9. Burnight, E.R., Gupta, M., Wiley, L.A., Anfinson, K.R., Tran, A., Triboulet, R., Hoffmann, J.M., Klaahsen, D.L., Andorf, J.L., Jiao, C., et al. (2017). Using CRISPR-Cas9 to Generate Gene-Corrected Autologous iPSCs for the Treatment of Inherited Retinal Degeneration. Mol. Ther. *25*, 1999–2013.
10. Collin, R.W.J., and Garanto, A. (2017). Applications of antisense oligonucleotides for the treatment of inherited retinal diseases. Curr. Opin. Ophthalmol. *28*, 260–266.
11. Carvalho, L.S., Turunen, H.T., Wassmer, S.J., Luna-Velez, M.V., Xiao, R., Bennett, J., and Vandenberghe, L.H. (2017). Evaluating Efficiencies of Dual AAV Approaches for Retinal Targeting. Front. Neurosci. *11*, 503.
12. Berger, A., Maire, S., Gaillard, M.C., Sahel, J.A., Hantraye, P., and Bemelmans, A.P. (2016). mRNA trans-splicing in gene therapy for genetic diseases. Wiley Interdiscip. Rev. RNA *7*, 487–498.
13. Trapani, I., Colella, P., Sommella, A., Iodice, C., Cesi, G., de Simone, S., Marrocco, E., Rossi, S., Giunti, M., Palfi, A., et al. (2014). Effective delivery of large genes to the retina by dual AAV vectors. EMBO Mol. Med. *6*, 194–211.
14. Konarska, M.M., Padgett, R.A., and Sharp, P.A. (1985). Trans splicing of mRNA precursors in vitro. Cell *42*, 165–171.
15. Solnick, D. (1985). Trans splicing of mRNA precursors. Cell *42*, 157–164.
16. Chao, H., Mansfield, S.G., Bartel, R.C., Hiriyanna, S., Mitchell, L.G., Garcia-Blanco, M.A., and Walsh, C.E. (2003). Phenotype correction of hemophilia A mice by spliceosome-mediated RNA trans-splicing. Nat. Med. *9*, 1015–1019.
17. Berger, A., Lorain, S., Joséphine, C., Desrosiers, M., Peccate, C., Voit, T., Garcia, L., Sahel, J.A., and Bemelmans, A.P. (2015). Repair of rhodopsin mRNA by spliceosome-mediated RNA trans-splicing: a new approach for autosomal dominant retinitis pigmentosa. Mol. Ther. *23*, 918–930.
18. Koller, U., Wally, V., Mitchell, L.G., Klausegger, A., Murauer, E.M., Mayr, E., Gruber, C., Hainzl, S., Hintner, H., and Bauer, J.W. (2011). A novel screening system improves genetic correction by internal exon replacement. Nucleic Acids Res. *39*, e108.
19. Puttaraju, M., Jamison, S.F., Mansfield, S.G., Garcia-Blanco, M.A., and Mitchell, L.G. (1999). Spliceosome-mediated RNA trans-splicing as a tool for gene therapy. Nat. Biotechnol. *17*, 246–252.
20. Mansfield, S.G., Kole, J., Puttaraju, M., Yang, C.C., Garcia-Blanco, M.A., Cohn, J.A., and Mitchell, L.G. (2000). Repair of CFTR mRNA by spliceosome-mediated RNA trans-splicing. Gene Ther. *7*, 1885–1895.
21. Liu, X., Luo, M., Zhang, L.N., Yan, Z., Zak, R., Ding, W., Mansfield, S.G., Mitchell, L.G., and Engelhardt, J.F. (2005). Spliceosome-mediated RNA trans-splicing with recombinant adeno-associated virus partially restores cystic fibrosis transmembrane conductance regulator function to polarized human cystic fibrosis airway epithelial cells. Hum. Gene Ther. *16*, 1116–1123.
22. Liu, X., Jiang, Q., Mansfield, S.G., Puttaraju, M., Zhang, Y., Zhou, W., Cohn, J.A., Garcia-Blanco, M.A., Mitchell, L.G., and Engelhardt, J.F. (2002). Partial correction of endogenous DeltaF508 CFTR in human cystic fibrosis airway epithelia by spliceosome-mediated RNA trans-splicing. Nat. Biotechnol. *20*, 47–52.
23. Monjaret, F., Bourg, N., Suel, L., Roudaut, C., Le Roy, F., Richard, I., and Charton, K. (2014). Cis-splicing and translation of the pre-trans-splicing molecule combine with efficiency in spliceosome-mediated RNA trans-splicing. Mol. Ther. *22*, 1176–1187.
24. Trochet, D., Prudhon, B., Jollet, A., Lorain, S., and Bitoun, M. (2016). Reprogramming the Dynamin 2 mRNA by Spliceosome-mediated RNA Trans-splicing. Mol. Ther. Nucleic Acids *5*, e362.
25. Lorain, S., Peccate, C., Le Hir, M., Griffith, G., Philippi, S., Précigout, G., Mamchaoui, K., Jollet, A., Voit, T., and Garcia, L. (2013). Dystrophin rescue by trans-splicing: a strategy for DMD genotypes not eligible for exon skipping approaches. Nucleic Acids Res. *41*, 8391–8402.
26. Mearini, G., Stimpel, D., Krämer, E., Geertz, B., Braren, I., Gedicke-Hornung, C., Précigout, G., Müller, O.J., Katus, H.A., Eschenhagen, T., et al. (2013). Repair of Mybpc3 mRNA by 5′-trans-splicing in a Mouse Model of Hypertrophic Cardiomyopathy. Mol. Ther. Nucleic Acids *2*, e102.
27. He, X., Liu, F., Yan, J., Zhang, Y., Yan, J., Shang, H., Dou, Q., Zhao, Q., and Song, Y. (2015). Trans-splicing repair of mutant p53 suppresses the growth of hepatocellular carcinoma cells in vitro and in vivo. Sci. Rep. *5*, 8705.
28. He, X., Liao, J., Liu, F., Yan, J., Yan, J., Shang, H., Dou, Q., Chang, Y., Lin, J., and Song, Y. (2015). Functional repair of p53 mutation in colorectal cancer cells using trans-splicing. Oncotarget *6*, 2034–2045.
29. Wally, V., Brunner, M., Lettner, T., Wagner, M., Koller, U., Trost, A., Murauer, E.M., Hainzl, S., Hintner, H., and Bauer, J.W. (2010). K14 mRNA reprogramming for dominant epidermolysis bullosa simplex. Hum. Mol. Genet. *19*, 4715–4725.
30. Uchida, N., Washington, K.N., Mozer, B., Platner, C., Ballantine, J., Skala, L.P., Raines, L., Shvygin, A., Hsieh, M.M., Mitchell, L.G., and Tisdale, J.F. (2017). RNA Trans-Splicing Targeting Endogenous β-Globin Pre-Messenger RNA in Human Erythroid Cells. Hum. Gene Ther. Methods *28*, 91–99.
31. Murauer, E.M., Koller, U., Hainzl, S., Wally, V., and Bauer, J.W. (2013). A reporter-based screen to identify potent 3′ trans-splicing molecules for endogenous RNA repair. Hum. Gene Ther. Methods *24*, 19–27.
32. Tockner, B., Kocher, T., Hainzl, S., Reichelt, J., Bauer, J.W., Koller, U., and Murauer, E.M. (2016). Construction and validation of an RNA trans-splicing molecule suitable to repair a large number of COL7A1 mutations. Gene Ther. *23*, 775–784.
33. Puttaraju, M., DiPasquale, J., Baker, C.C., Mitchell, L.G., and Garcia-Blanco, M.A. (2001). Messenger RNA repair and restoration of protein function by spliceosome-mediated RNA trans-splicing. Mol. Ther. *4*, 105–114.
34. Goujon, M., McWilliam, H., Li, W., Valentin, F., Squizzato, S., Paern, J., and Lopez, R. (2010). A new bioinformatics analysis tools framework at EMBL-EBI. Nucleic Acids Res. *38*, W695–W699.
35. Sievers, F., Wilm, A., Dineen, D., Gibson, T.J., Karplus, K., Li, W., Lopez, R., McWilliam, H., Remmert, M., Söding, J., et al. (2011). Fast, scalable generation of high-quality protein multiple sequence alignments using Clustal Omega. Mol. Syst. Biol. *7*, 539.
36. National Library of Medicine. (2017). NCBI Nucleotide BLAST: Search nucleotide databases using a nucleotide query. https://blast.ncbi.nlm.nih.gov/Blast.cgi?PAGE_TYPE=BlastSearch.
37. Seth, P., Miller, H.B., Lasda, E.L., Pearson, J.L., and Garcia-Blanco, M.A. (2008). Identification of an intronic splicing enhancer essential for the inclusion of FGFR2 exon IIIc. J. Biol. Chem. *283*, 10058–10067.

38. Lorain, S., Peccate, C., Le Hir, M., and Garcia, L. (2010). Exon exchange approach to repair Duchenne dystrophin transcripts. PLoS ONE *5*, e10894.

39. Bennicelli, J.L., Vassireddy, V., and Bennett, J. (2012). 430. CEP290 Minigene Model of Common Splice Site Mutation in Leber Congenital Amaurosis. Mol. Ther. *20* (*Suppl 1*), S167–S168.

40. Dalkara, D., Byrne, L.C., Kilmczak, R.R., Visel, M., Yin, L., Merigan, W.H., Flannery, J.G., and Schaffer, D.V. (2013). vivo-directed evolution of a new adeno-associated virus for therapeutic outer retinal gene delivery from the vitreous. Sci. Transl. Med *5*, 189ra76.

41. Gruber, C., Koller, U., Murauer, E.M., Hainzl, S., Hüttner, C., Kocher, T., South, A.P., Hintner, H., and Bauer, J.W. (2013). The design and optimization of RNA trans-splicing molecules for skin cancer therapy. Mol. Oncol. *7*, 1056–1068.

42. Philippi, S., Lorain, S., Beley, C., Peccate, C., Précigout, G., Spuler, S., and Garcia, L. (2015). Dysferlin rescue by spliceosome-mediated pre-mRNA trans-splicing targeting introns harbouring weakly defined 3′ splice sites. Hum. Mol. Genet. *24*, 4049–4060.

43. Cooper, T.A. (2005). Use of minigene systems to dissect alternative splicing elements. Methods *37*, 331–340.

44. Garanto, A., van Beersum, S.E., Peters, T.A., Roepman, R., Cremers, F.P., and Collin, R.W. (2013). Unexpected CEP290 mRNA splicing in a humanized knock-in mouse model for Leber congenital amaurosis. PLoS ONE *8*, e79369.

45. Shimada, H., Lu, Q., Insinna-Kettenhofen, C., Nagashima, K., English, M.A., Semler, E.M., Mahgerefteh, J., Cideciyan, A.V., Li, T., Brooks, B.P., et al. (2017). In Vitro Modeling Using Ciliopathy-Patient-Derived Cells Reveals Distinct Cilia Dysfunctions Caused by CEP290 Mutations. Cell Rep. *20*, 384–396.

46. Yu, W., Mookherjee, S., Chaitankar, V., Hiriyanna, S., Kim, J.W., Brooks, M., Ataeijannati, Y., Sun, X., Dong, L., Li, T., et al. (2017). Nrl knockdown by AAV-delivered CRISPR/Cas9 prevents retinal degeneration in mice. Nat. Commun. *8*, 14716.

OMTN, Volume *12*

Supplemental Information

Spliceosome-Mediated Pre-mRNA

trans-Splicing Can Repair *CEP290* mRNA

Scott J. Dooley, Devin S. McDougald, Krishna J. Fisher, Jeanette L. Bennicelli, Lloyd G. Mitchell, and Jean Bennett

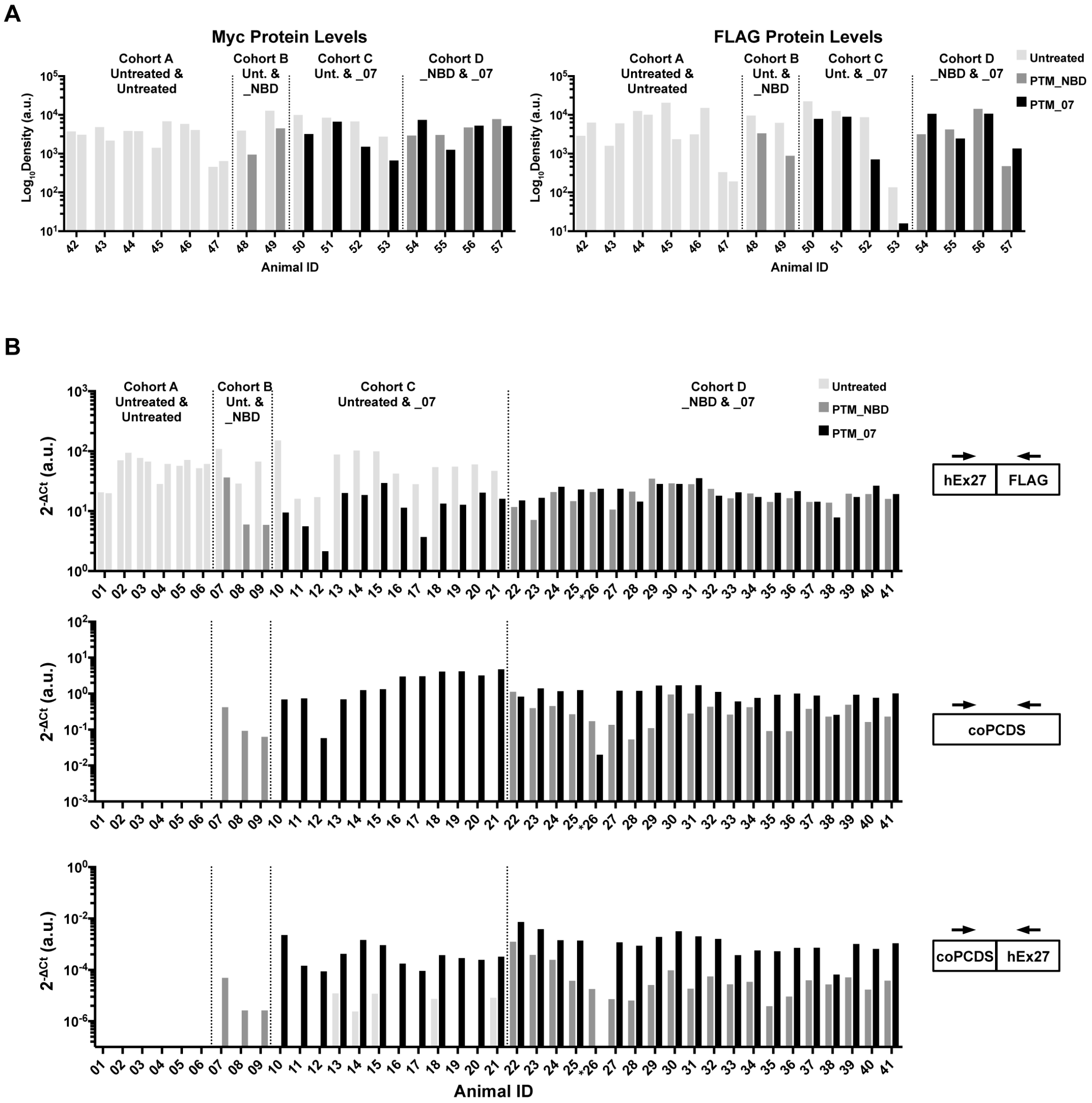

Figure S1. Individual treated murine samples by contralateral eye.

(A) Densitometry quantification of western blot images of Myc and FLAG at 24.3 kDa from protein extracts of whole eyes of mini-*CEP290* mice. Eyes were treated by sub-retinal injection with 5’ PTMs encoded in AAV serotype 7m8 capsids. Samples are grouped by contralateral eye treatments per animal and treatment cohorts are separated by vertical dashed lines. Values were standardized to α-Tubulin and logarithm values were utilized to compare substantially different values between mice. A.U., arbitrary units.

(B) Quantitative PCR from cDNA generated by RNA extracts from whole eyes of mini-*CEP290* mice. TaqMan probes were designed to the junctions of *homo sapiens* exon 27 and FLAG to detect total expression of the mini-gene (*top*), to a region within the PCDS to detect total expression of the PTM (*center*), or to the novel junction of codon-optimized *CEP290* PCDS and *Homo sapiens CEP290* exon 27 (*bottom*). Injection of animal 26 with PTM_07, indicated by asterisk (*) did not yield a visible bleb and concurrently no *trans*-splicing was detected in this sample. Samples are grouped by contralateral eye treatments per animal and treatment cohorts are separated by vertical dashed lines. Ct values were standardized to the β-2-microglobin control gene to generate ΔCt values. A.U., arbitrary units.
